# Autism Spectrum Disorders: Analysis of Mobile Elements at 7q11.23 Williams–Beuren Region by Comparative Genomics

**DOI:** 10.3390/genes12101605

**Published:** 2021-10-12

**Authors:** Francesca Anna Cupaioli, Chiara Fallerini, Maria Antonietta Mencarelli, Valentina Perticaroli, Virginia Filippini, Francesca Mari, Alessandra Renieri, Alessandra Mezzelani

**Affiliations:** 1 Institute of Biomedical Technologies, Italian National Research Council, Via Fratelli Cervi 93, 20090 Segrate, Italy; francesca.cupaioli@itb.cnr.it; 2 Med Biotech Hub and Competence Center, Department of Medical Biotechnologies, University of Siena, 53100 Siena, Italy; fallerini2@unisi.it (C.F.); valentina.perticaroli@dbm.unisi.it (V.P.); virginia.filippini@dbm.unisi.it (V.F.); francesca.mari@unisi.it (F.M.); alessandra.renieri@unisi.it (A.R.); 3 Medical Genetics, University of Siena, 53100 Siena, Italy; 4 Genetica Medica, Azienda Ospedaliero Universitaria Senese, 53100 Siena, Italy; mariaantonietta.mencarelli@unisi.it

**Keywords:** transposable elements, Williams–Beuren syndrome, dog sociability, comparative genomics, 7q11.23, indel, hyper-methylated, sociability, dosage sensitive genes, genetic variants

## Abstract

Autism spectrum disorders (ASD) are a group of complex neurodevelopmental disorders, characterized by a deficit in social interaction and communication. Many genetic variants are associated with ASD, including duplication of 7q11.23 encompassing 26–28 genes. Symmetrically, the hemizygous deletion of 7q11.23 causes Williams–Beuren syndrome (WBS), a multisystem disorder characterized by “hyper-sociability” and communication skills. Interestingly, deletion of four non-exonic mobile elements (MEs) in the “canine WBS *locus*” were associated with the behavioral divergence between the wolf and the dog and dog sociability and domestication. We hypothesized that indel of these MEs could be involved in ASD, associated with its different phenotypes and useful as biomarkers for patient stratification and therapeutic design. Since these MEs are non-exonic they have never been discovered before. We searched the corresponding MEs and *loci* in humans by comparative genomics. Interestingly, they mapped on different but ASD related genes. The *loci* in individuals with phenotypically different autism and neurotypical controls were amplified by PCR. A sub-set of each amplicon was sequenced by Sanger. No variant resulted associated with ASD and neither specific phenotypes were found but novel small-scale insertions and SNPs were discovered. Since MEs are hyper-methylated and epigenetically modulate gene expression, further investigation in ASD is necessary.

## 1. Introduction

Autism spectrum disorders (ASD) and Williams–Beuren syndrome (WBS) are two conditions that manifest symmetrically opposite behavioral phenotypes [1].

ASD are a group of neurodevelopmental disorders that include a wide range of phenotypes characterized by a deficit in social interactions, verbal and non-verbal communication, restricted interests, and repetitive behaviors (www.who.int; https://www.cdc.gov/, accessed on 25 June 2021). Sensory abnormalities, including auditory sensitivity, are also common among individuals with autism [2]. ASD affects more males than females (males:females = 4:1) and cases have been dramatically increasing since the last decades reaching the prevalence of one in 54 in the United States [3]. There is no cure for ASD, although early diagnosis coupled with early and personalized interventions can significantly improve the outcome [4].

**Figure 1:**
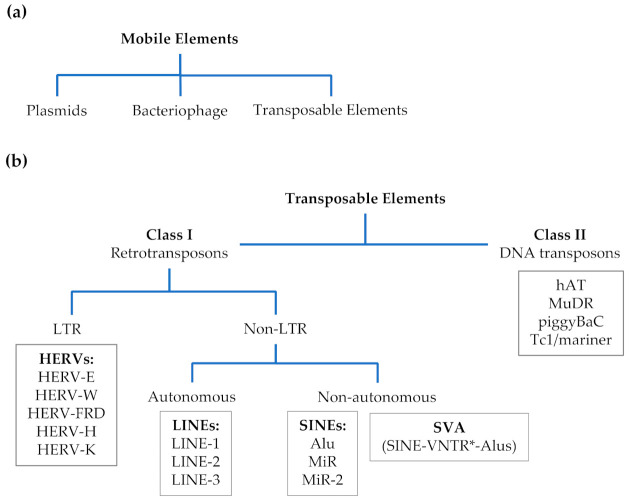
Mobile elements (MEs) and transposable elements (TEs) in human. (**a**) human MEs, (**b**) classification of human TEs. * VNTR = Variable Number Tandem Repeat.

ASD has a complex and uncertain etiology, mainly due to gene-environment interaction. As for the environment, endocrine disruptors and intestinal microbes are getting a great attention from the scientific community [5,6,7]. Although genetics plays a key role in ASD etiology and hundreds of causative genetic and risk factors have been described, only 25–30% of patients carry genetic variants [8]. Despite the large number of genes involved, most share the same function converging to and affecting a small number of biological processes, especially synaptic functioning, epigenetic factors, and regulators of calcium channel activity [9]. Moreover, genetic modulators, such as epigenetic factors and copy number variants (CNVs), modulate the penetrance of risk genes, leading to different phenotypes for similar variants [10]. Then again, the stability of autism-risk genes can be related to the content of transposable element (TE) [11]. TEs belong to the mobile elements (MEs) and are DNA sequences able to move around the genome often affecting the activity of including or neighboring genes by altering their genomic sequence and structure, and exerting epigenetic effects [12]. It has been estimated that over two-thirds of the human genome results from MEs with evolutionarily remarkable functions [13]. Human MEs classification is represented in Figure 1.

WBS (OMIM #194050) is a rare (1 in 7500–10,000 individuals) multisystem disorder that manifests a phenotype that is the opposite to ASD in the social domain. Indeed, it exhibits “hyper-sociability” due to language skills and loquaciousness as well as empathetic and sociable behavior [14]. Unlike ASD, the etiology of WBS is well known: it is genetically determined by the hemizygous and heterogeneous deletion at 7q11.23 region, called WBS *locus,* that encompass 26–28 genes [15]. This region is susceptible to recurrent chromosomal rearrangements [16], and its symmetric duplication (7dupASD) (#609757) is one of the genetic variants strongly associated with a severe delay in verbal communication and ASD [17]. This suggests that genes at WBS *locus* are involved in human language and communication and their effects depend on WBS gene CNVs [1,18]. Indeed, Adamo and colleagues demonstrated that 7q11.23 dosage imbalance modulates transcriptional processes in disease-relevant transcriptional programs causing symmetrically opposite modifications in human pluripotent stem cells models of 7dupASD and WBS. In particular, the gene dosage of *general transcription factor IIi* (*GTF2I, alias WBSCR17*) at 7q11.23 strictly corresponds to mRNA and protein level. *GTF2I*, is part of a complex that exerts epigenetic effects that are crucial for neurodevelopment [1]. In a recent study, three compounds were able to epigenetically inhibit the abnormal mRNA and protein level of *GTF2I* in 7DupASD patient-derived cell models thus representing promising therapeutic results [19].

Interestingly, some discerning studies about the genetic basis of behavioral characteristics of dogs (i.e., sociability, curiosity, playfulness, aggression, and separation anxiety) associated with their personality and functionally breed differences [20] highlighted the role of structural variants (SVs) in the predisposition to domestication. Specifically, CNVs of TEs were found in the genomic region corresponding to the “canine WBS susceptible *locus*” on chromosome 6 (CFA6). In particular, in the dog, SVs in two *GTF2I* family genes, both involved in humans in WBS, are associated with extreme and stereotypical hypersociability (evaluated by standard behavioral assays) and discriminated between dog and wolf [21]. TEs insertion determines dogs’ inclination to prolonged social contact, thus suggesting that the WBS *locus* is involved in dogs hypersociability that, in turn, is the central element of their domestication [21,22]. This also suggests that TEs at the canine WBS *locus* can function as epigenetic modulators for social behavior at the canine WBS *locus*. Indeed, a subsequent study about dog behavior proved that MEs, that map within non-coding sequences of canine WBS *locus*, decreased the expression of some genes involved in WBS, including *GTF2I* [22,23]. MEs are notably hyper-methylated so they can epigenetically silence flanking genes [24]. Furthermore, variations in TEs insertions, in or near *Polypeptide N-Acetylgalactosaminyltransferase 17* (*GALNT17*, *alias WBSCR17*) at Cfa6.6 and Cfa6.7 *loci* as well as *GTF2I* at Cfa6.66 *locus* significantly associated with a heightened propensity to initiate prolonged social contact or hyper-sociability [22].

Since WBS *locus* is central for modulating sociability in dogs and humans and in case of dogs, TEs in this genomic region can modulate the expression of the genes included, we hypothesized that: (i) ME variants involved in dog domestication could affect the human WBS region and be involved in ASD or associated with specific ASD phenotypes (ii) in human, these MEs could map in non-coding regions and have never been detected so far because most genomic studies on ASD consist in exome sequencing and genome wide analysis (GWAS) [25].

The aim of this study was to find in humans a possible association between variants of the MEs detected in Cfa6.6, Cfa6.7, and Cfa6.66 *loci* [21,22] and ASD or ASD specific phenotypes. These variants could be useful as biomarkers for early diagnosis, patient stratification and as targets for future personalized therapeutic interventions.

To this purpose we first searched the sequences corresponding to these canine *loci* in the human genome by bioinformatics comparative genomics and identified the MEs; then, we tested the presence of possible ME indels in a cohort of individuals with phenotypically different ASD and neurotypical controls by PCR DNA amplification.

No variant was found associated with ASD nor with specific phenotypes, but novel small-scale insertions and SNPs were found in a sub-group of sequenced amplicons. In ASD, possible variants of TEs at 7q11.23 need further investigations.

## 2. Materials and Methods

### 2.1. Comparative Genomic Analysis

**Figure 2:**
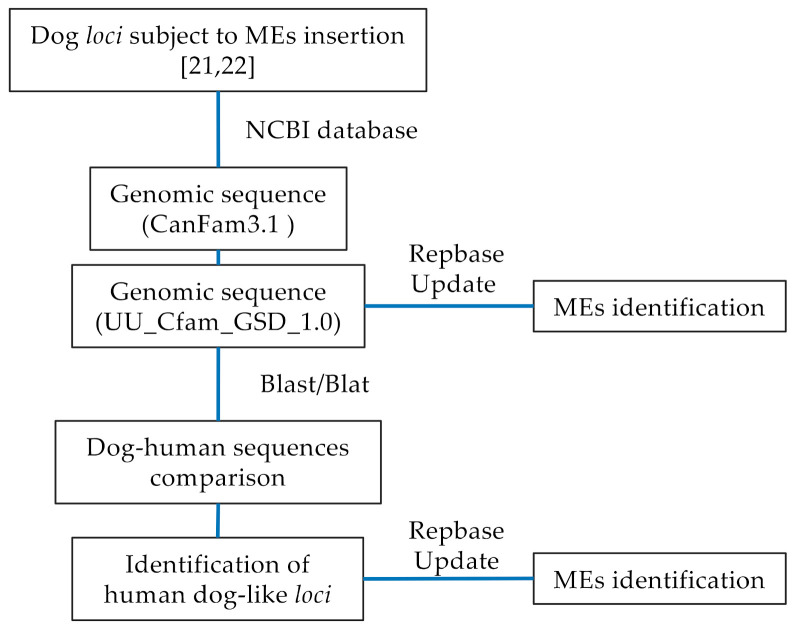
Workflow to identify human dog-like *loci* involved in domestication and subjected to the insertion of MEs.

We initially downloaded from the NCBI database (https://www.ncbi.nlm.nih.gov/genome/gdv/, accessed on 15 March 2021), the genomic sequences of the canine WBS *locus* that include MEs insertion; they belong to *WBSCR17* (Cfa6.6 and Cfa6.7) and *GTF2I* (Cfa6.66) genes [21,22]. The original paper [21,22] refers to the *Canis lupus familiaris* (dog) genome assembly CanFam3.1, so we reported the sequences to the latest release of the dog reference genome, assembly UU_Cfam_GSD_1.0 [26]. Then, we aligned the dog genomic sequences with the human reference genome, assembly GRCh38.p13, by sequence alignment tools (https://genome.ucsc.edu, accessed on 16 March 2021; https://blast.ncbi.nlm.nih.gov/Blast.cgi, accessed on 22 March 2021) to perform dog–human sequence comparison and identify the human *loci* corresponding to those of the dog (Figure 2).

The MEs identification tools CENSOR (https://www.girinst.org/censor/index.php, accessed on 13 May 2021) and RepeatMasker (by _UCSC), based on Repbase Update repeat library from the Genetic Information Research Institute (GIRI), were used to screen MEs in dog and human sequences.

### 2.2. MEs Detection in ASD Patients with ASD and Neurotypical Controls

#### 2.2.1. Subjects

The University of Siena recruited 94 subjects with ASD (80 males (85.1%) and 14 females (14.9%)) and 76 neurotypical controls (36 males (47.4%) and 40 females (52.6%)). The diagnoses were: 59 ASD (62.7%), 14 Intellectual Disability (ID) (14.8%), eight Pervasive developmental disorder (8.5%), eight psychomotor retardation (PMR) (8.5%), one behavior disorder (1.1%), one ASD and PMR (1.1%), one ASD and ataxia (1.1%), one ID and seizures (1.1%), one ID and one Asperger’s syndrome (1.1%).

#### 2.2.2. Diagnosis

Subject diagnoses involved in the study were performed according to the DSM-IV TR (American Psychiatric Association, 2000) for patients evaluated before 2013 and by DSM-5 (American Psychiatric Association, 2013) for those who had access to genetic counselling later, or by the ADOS-2 [27] and ADI-R [28].

#### 2.2.3. Samples 

Genomic DNA samples collected from the same subjects were provided by Telethon Network of Genetic Biobanks (Cell line and DNA bank of Rett Syndrome, X-linked mental retardation and other genetic diseases, member of the Telethon Network of Genetic Biobanks (project no. GTB18001).

#### 2.2.4. Primer Design

PCR primers were designed to amplify the human *loci*, including the previously identified MEs, by Primer3 (https://primer3.ut.ee, accessed on 12 April 2021). The sequences of the primers are reported in Table 1.

#### 2.2.5. PCR

The genomic DNA (20 ng) of 94 subjects with ASD and 76 neurotypical controls were amplified in 10 μL of reaction mixture (Platinum™ SuperFi™ PCR Master Mix, Thermo Fisher Scientific, Wilmington, DE, USA) as suggested by the supplier. The PCR conditions were set as suggested by the manufacturer and based on primer characteristics, as reported in Table 1. In particular, the extension time was prolonged to amplify up to 3 kb, thus the possible indel within MEs could also be amplified. To detect the presence of MEs indel, the PCR products were run on 2% or 1% pre-cast agarose gel (E-Gel agarose gels, Invitrogen™, Thermo Fisher Scientific, Wilmington, DE, USA) for products <1000 pb and >1000 bp, respectively.

#### 2.2.6. Sanger Sequencing

Twenty PCR products for each amplified *locus* were purified (CleanSweep PCR Purification Reagent, Applied Biosystems, Thermo Fisher Scientific, Wilmington, DE, USA) and sequenced by Sanger method using both forward and reverse PCR primers (Table 1). The amplicons were obtained from 10 individuals with ASD (one female with autistic disorder and one with ID; six males with autistic disorder and two with ID) and 10 from neurotypical controls (two females and eight males).

#### 2.2.7. Analysis of Genetic Variations

To confirm the specificity of the PCR products and to detect possible variants, the Sanger sequences of amplified human *loci* were aligned to those of human reference genes (GRCh38.p13; Gene ID: 64409, *GALNT17*; ID: 9369, *NRXN3*; upstream of ID: 10251, *SPRY3*; ID: 106481735, *GTF2IP14*; ID: 100420546, *GTF2IP5*; ID: 84679, *SLC9A7*). SNPs, microsatellites, small-scale insertions and deletions were verified by dbSNPs database (https://www.ncbi.nlm.nih.gov/snp/, accessed on 5 July 2021). Fisher’s exact test was applied to each variation identified to detect significant differences between patients with ASD and controls. Consensus sequences were obtained for the sequenced *loci* by Unipro UGENE software, and the identity of consensus from patients with ASD and neurotypical controls evaluated.

## 3. Results

### 3.1. Dog–Human Sequence Correspondence

We identified the sequence of the repeated regions belonging to *GALNT17* (Cfa6.6 and Cfa6.7) and *GTF2I* (Cfa6.66) *loci* and relative MEs (Table 2). Comparison of canine sequences to human sequences showed similarity for all investigated *loci (*Table 3).

The canine cfa6.6 in *GALNT17* (chr6:2,441,471-2,442,028, UU_Cfam_GSD_1.0/CanFam4) maps in human genome (GRCh38.p13) on *GALNT17* at position chr7:71,198,601–71,198,849 with score = 100 and identity of 75.2%. The dog Cfa6.6 includes the TEs: MER5A (family hAT-Charlie, class DNA) at chr6:2,441,321–2,441,475; (CA)n (family Simple_repeat, class Simple_repeat) at chr6:2,441,548–2,441,589; SINEC2A1_CF (family tRNA, class SINE) at chr6:2,441,653–2,441,841. The corresponding region in *Homo sapiens* does not contain repeating elements.

Cfa6.7, at chr6: 2,466,130–2,466,636 (Cfam_GSD_1.0/CanFam4) is a second repeated region identified in canine *GALNT17* [21,22,23]. Dog Cfa6.7 includes the repeating elements MER21C (Family ERVL, Class: LTR) at position chr6:2,465,886-2,466,144 and chr6:2,466,179–2,466,334, and the SINEC2A1_CF (family tRNA, class SINE) at chr6:2,466,362–2,466,558. This amplicon does not align with *GALNT17* in the human genome, but does align, with low score, in more than one genomic region. Moreover, the UCSC (Blat) and NCBI (Blast) return different results. Between these two regions, we focused on results including a gene with the highest score and identity. Interestingly, dog Cfa6.7 aligns with human *neurexin* (*NRXN3*) (Chr14:79,081,617–79,081,678) with 11% of coverage and 78% of identity. In *Homo sapiens*, the aligned region straddles the repeating elements AluSc8 (Family Alu, Class: SINE, chr14:79,081,335–79,081,634) and contains the TE L2c (Family L2, Class LINE, chr14:79,081,635–79,081,755).

The Cfa6.7 sequence also aligns with human *Solute Carrier Family 9 Member A7* (*SLC9A7*) at position ChrX:46,624,294–46,624,350 with score = 43 and identity = 93%. This region is in the TE MER21C (Family ERVL, Class LTR, chrX:46,623,722–46,624,477). Furthermore, Cfa6.7 amplicon aligns also with human (chrX:155,795,613–155,795,656), about 15 kb over the *Sprouty RTK Signaling Antagonist 3 (SPRY3)* gene (chrX:155,612,586–155,782,459), with score = 32 and identit y = 94.5%. This region is likewise in the TE MER21C (Family ERVL, Class LTR), at chrX:155,795,271–155,795,776.

The Cfa6.66 amplicon has a dissimilar sequence depending on genome release: chr6:5,753,657–5,754,161 (CanFam3.1), 505 bp in length, and chr6:5,671,518–5,671,763 (Cfam_GSD_1.0/CanFam4), 246 bp. Nonetheless, these regions belong to *GTF2I*. The CamFam3.1 reports that the region includes two repeating elements: an AT_rich (family Low_complexity, Class Low_complexity) at position chr6:5,753,732–5,753,757, and a SINEC_Cf (Family tRNA-Lys, Class SINE) at position chr6:5,753,797–5,753,983. However, the sequence reported in fam_GSD_1.0/CanFam4 does not contain MEs. The sequence reported in the most recent release of dog genome, CanFam4, of Cfa6.66 aligns in *Homo sapiens* to Chr7:65,095,911–65,096,110 (*GTF2IP14*) and chr7:65,785,354–65,785,553 (*GTF2IP5*) in both cases with 76% of identity. These two regions have 99% of identity. The region in *GTF2IP14* includes the TEs L1MC5a (LINE/L1) at position Chr7:65,095,820–65,095,976. No ME is present in the interested region of *GTF2IP5*.

### 3.2. PCR Results

**Figure 3:**
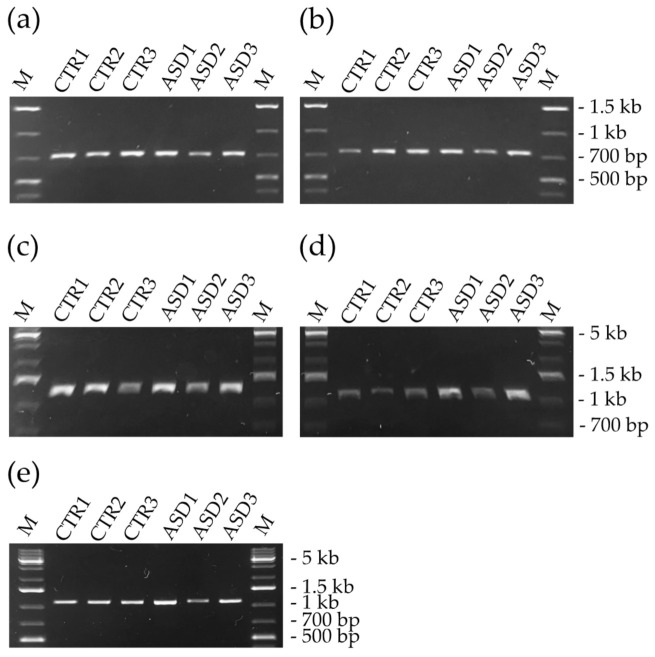
Agarose gel electrophoresis. (**a**) amplicons of *GALNT17* (716 bp) and (**b**) *NRNX3* (738 bp); (**c**) amplicons of *GTF2IP14* (1249 bp) and (**d**) *SPRY3* (1114 bp); (**e**) amplicons of *SLC9A7* (1089 bp). Amplicons of the different *loci* belong to the same three neurotypical controls and three patients suffering from ASD. No differences between patients and controls were detected within the same *locus*. (**a**,**b**) have the same 1 Kb marker; (**c**,**d**) have the same 1 Kb marker. Abbreviation: CTR, control; ASD, autism spectrum disorders; M, marker.

Genomic DNA of 94 individuals with ASD and 76 neurotypical controls were successfully amplified by PCR for all the tested *loci*. Agarose gel electrophoresis of amplicons did not detect any difference in length between patients and controls indicating that no indel was present in the tested genes (Figure 3).

### 3.3. Sanger Sequencing Results

The alignments of the amplicon sequences obtained by the Sanger method confirmed that the PCR products were those targeted. The identity of consensus sequences between patients with ASD and neurotypical controls was higher or equal to 95% for all tested human *loci* (Table 3). No indel larger than three bases were observed in DNA sequences of patients with ASD nor in neurotypical controls when compared with reference genome, and several variants were identified and listed in detail in Table 3.

In the amplicons *GALNT17* (Cfa6.7) two common SNPs (rs1202647 and rs10260271) were identified as equally distributed in patients and controls.

No SNPs, microsatellites, small-scale insertions, and deletions were found in the amplicons targeted in *NRNX3* (Cfa6.7).

In the amplicons targeted about 15 kb upstream the *SPRY3* gene, three novel SNPs were identified in one patient out of 10 as well as one novel SNP in one neurotypical control out of 10.

In *SLC9A7* (Cfa6.7), one novel SNP was found in one individual with ASD while the rare SNP rs1238052830 and three novel SNPs was found in one control.

The consensus of *GTF2I* (Cfa6.66) amplicons aligns to chr7:65,095,528–65,096,623 in *GTF2IP14* (identity = 99.5%), and to Chr7:65,784,975–65,786,076 in *GTF2IP5* (identity = 98.5%). One of the main differences between *GTF2IP14* and *GTF2IP5* is the insertion of the oligos AAAA and CAAAAA in *GTF2IP5* within the interval chr765,785,905–65,785,928. Since the sequenced amplicons did not include these two insertions, we probably amplified *GTF2IP14*. A total of 34 variants were identified: three common SNPs (rs1799101, rs10262238, rs71562961) and 10 novel SNPs, equally distributed within ASD and neurotypical groups; three new small-scale insertions and 13 novel SNPs, each in a single subject with ASD; and five novel SNPs, each in a single neurotypical individual.

No significant differences (*p* > 0.05) were found between patients and controls in each identified variant.

## 4. Discussion

WBS *locus* at 7q11.23, including 26–28 genes, is involved in the human social domain and the expression of its genes is modulated in a dosage dependent mode. Indeed 7q11.23 duplication is associated with language problems and is one of the many genetic variants linked to ASD characterized by deficit in communication and social interaction. On the contrary, its hemizygous deletion causes WBS, a multisystem disorder with opposite behavior: loquaciousness and hyper-sociability.

Interestingly, the insertion of three non-exonic MEs, in the “canine WBS *locus*” decreases the expression of their flanking genes and is associated to dog sociability and domestication. Genetic studies regarding dog behavior are improving and associated with the scores obtained by behavioral tests that specifically define social and attitudinal skills as well as personality traits [20,21,22]. Since WBS *locus* is involved in social behavior in dogs and humans, we translated the known knowledge from the genetics of dog sociability to human by means of a comparative genomic study.

In ASD, the rate and role of variants in noncoding regions, including structural variations, indels and SNPs are probably limited [29] as well as MEs in coding sequences, which are poorly studied [30]. Nevertheless, the role of TEs in psychiatric disorders is emerging and transposomic technologies are developing [31,32,33].

Here, for the first time, we used a comparative genomic approach to verify if MEs in human WBS *locus* and their possible variants could be associated with ASD or its specific phenotypes, as happens in canine WBS *locus*. Moreover, these MEs maps in non-coding regions may have escaped the discovery; indeed, most of the genetic studies about ASD genetics focus on exome sequencing and GWAS [25].

We first searched the ME dog–human sequences correspondence by comparative genomic analysis. We found that the canine MEs in *cfa6.6* maps to the corresponding *GALNT17* gene of the human genome, and caf6.66, in canine *GTF2I* gene, to two human *GTF2I* pseudogenes. On the contrary to our expectations, dog *cfa6.7* that also maps in *GALNT17,* aligns with three different human genes *(NRXN3, SLC9A7* and 15 kb upstream *SPRY3)* that are already involved in autism, two of which are X-linked. This is in line with previous studies that found variants in genes both associated with dog personality traits and human neurologic conditions. For instance, in dogs, SNPs associated with noise fear, that often afflicts individuals with autism, map close to *calcium dependent secretion activator 2*. This gene acts in neurons and neuroendocrine cells modulating the exocytosis of synaptic and dense-core vesicles and, in humans, its variants may contribute to autism susceptibility [34,35].

Moreover, we found that the canine Cfa6.6 *locus* at *GALNT17* maps in the same gene of human genome reference at 7q11.22, chr7:71,198,601–71,198,849, but does not contain repeating elements nor carry any variants in length or sequence.

The *Cfa6.7*, also at canine *GALNT17*, aligns with three human genomic regions. The first one is the human *NRXN3* (Chr14:79,081,617–79,081,678) with 11% of coverage and 79% of identity. This human sequence straddles the repeating elements AluSc8 and includes the ME L2c. No variant was found in this region by Sanger sequencing but, interestingly, NRXN3 is a member of the NRXN family. NRXN3 localizes at presynapses and it is a cell surface receptor with a key role in neural circuit assembly and restructuring [36,37]. Of relevance, the neurexins-neuroligins pathway is one of the most affected pathways in ASD [38]. Indeed, mutations in NRXN family members, including *NRXN3*, have been repeatedly identified in ASD. The potential pathologic role(s) of these variants is still unclear and both loss- and gain-of-function, because of complex transcriptome changes, have been cited [39].

The second one corresponds to the human *SLC9A7* at position ChrX:46,624,294–46,624,350 with 43% of coverage and 93% of identity and includes a ME MER21C. Here we found one novel SNP in one individual in the spectrum (male, with ASD) as well as the rare SNP rs1238052830 and three novel SNPs in one control. *SLC9A7* encodes a sodium and potassium proton antiporter, its expression is enriched in the brain and, at the cellular level, the protein localizes in the trans-Golgi network and post-Golgi vesicles. Mutations in this gene have been reported in X-linked intellectual developmental disorder (MRX108; # 301024) [40] while variations in other *SLC9* family members are associated with autistic features [41].

The third one aligns 15 kb upstream *SPRY3* gene (chrX:155,612,586–155,782,459), with 32% of coverage and 94.5% of identity; this *locus* includes a ME, the MER21C. It maps at chrX:155,612,586–155,782,459 adjacent to *X-linked Trimethyllysine hydroxylase epsilon* gene, an ASD risk gene. SPRY inhibits neurite branching and complexity and high levels of the protein have been found in nervous system ganglion cells, especially cerebellar Purkinje cells that are deficient in autism [42,43]. Ning and colleagues [44] proposed that inadequate SPRY3 levels in these cells may interact with TrkB and p75NTR pathways leading to Purkinje cell pathology. Moreover, although *SPRY3* maps to the *second human X-Y pseudoautosomal region 2* (*PAR2*), the Y-linked allele is silenced. This makes the expression of the *SPRY3* X-linked allele crucial and *SPRY3* variants possible cohort of the male bias in autism prevalence. In this *locus* we discovered two novel SNPs in one male with ASD and two in two different controls.

The dog Cfa6.66, that belongs to *GTF2I* gene, aligns, in the human genome, at 7q11.21, to the *General Transcription Factor IIi Pseudogene 5* (*GTF2IP5)*, chr7:65,785,354–65,785,553, and 14 (*GTF2IP14)* at Chr7:65,095,911–65,096,110, in both cases with 76% of identity. These two regions share 99% of identity. Because of technical problems due to their very high homology, we amplified only *GTF2IP14* that includes the TEs L1MC5a (LINE/L1) at Chr7:65,095,820–65,095,976. As expected from a pseudogene, the *GTF2IP14* sequences carry a high number of variants: 34. Of these, three common SNPs (rs1799101, rs10262238, rs71562961) and 10 novel SNPs were found both in ASD and control group, while three new small-scale insertions and 13 novel SNPs only in ASD and five novel SNPs only in controls.

## 5. Conclusions

Many efforts have been devoted to unravel the complex genetics of ASD. Nevertheless, limited studies refer to TEs, a neglected type of SVs able to disrupt the architecture of a gene or to modulate its expression. Further insights into TEs implication in ASD should be carried out and could take advantage from comparative genomics. This original approach allows to study a genome, or a part of it, taking advantage of the knowledge developed on the genome of another species. In particular, many genetic markers associated with social behavior and measured by standard and reproducible behavioral tests are being obtained in the dog. Interestingly, these markers map in the same region as in humans and is associated with “sociability disorders”: ASD and WBS. Thus, they shed light on complex mechanisms of dog be but could also be translated to human. Moreover, possible indel of MEs associated with ASD or specific ASD phenotypes may be relevant to explain the pathogenic mechanisms of the condition and useful as biomarkers for patient stratification and targets for personalized interventions.

## Figures and Tables

**Table 1:** PCR and Sanger sequencing primers.

Dog locus	Human Gene	Primer (Human)	Oligo Sequence (Human)	Product Size	Anealing Condition (°C)
Cfa6.6	*GALNT17*	Forward	ACATGGTCCTTCGCTAGAGAGA	716	59.8
		Reverse	CCCCTTGGCCACCTAATCAA		
Cfa6.7	*NRXN3*	Forward	AGGTGCACATACTAAAACCAAATGA	738	59
		Reverse	ACTGTTTTGTCCTCATGTCTTTTCA		
	*SLC9A7*	Forward	ACCCGGCCAACCTCTATTCA	1089	60.5
		Reverse	GCCACATATCAGACACCATCCT		
	*SPRY3*	Forward	CCCGGCCAGCAGTTTGTTAT	1114	60.6
		Reverse	TGACTTGCTCCAGGTGATAATCTG		
Cfa6.66	*GTF2IP14*	Forward	CATCCCCGAACAGCATTAACA	1249	58.6
		Reverse	TGACCCATCATTACCAATCAGATTT		

**Table 2:** Dog–human sequence correspondence and relative TEs. The dog section highlights: the dog *loci* amplified by vonHoldt and colleagues [21,22], the genes they belong to, and their position in the dog genome assembly UU_Cfam_GSD_1; the TEs and their position detected in these *loci*. The human section reports the corresponding human dog-like *loci* identified by comparative genomic analysis, the TEs included in these regions and their position in human genome, assembly GRCh38.p13. Abbreviations: Chr, chromosome; TE, transposable element.

DOG (UU_Cfam_GSD_1.0)										Human (GRCh38.p13)								
Locus	Gene	Chr	Amplicon Start	Amplicon End	TE Name	TE Class/Family	Chr	TE Start	TE End	Gene	Chr	Amplicon Start	Amplicon End	TE Name	TE Class/Family	Chr	TE Start	TE End
cfa6_6	*GALNT17*	6	2,441,471	2,442,028	MER5A	DNA/hAT-Charlie	6	2,441,321	2,441,475	*GALNT17*	7	71,198,462	71,199,177					
					(CA)n	Simple_repeat	6	2,441,548	2,441,589									
					SINEC2A1_CF	SINE/tRNA	6	2,441,653	2,441,841									
cfa6_7	*GALNT17*	6	2,466,130	2,466,636	MER21C	LTR/ERVL	6	2,465,886	2,466,144	*NRXN3*	14	79,081,200	79,081,937	AluSc8	SINE/Alu	14	79,081,335	79,081,634
					MER21C	LTR/ERVL	6	2,466,179	2,466,334					L2c	LINE/L2	14	79,081,635	79,081,755
					SINEC2A1_CF	SINE/tRNA	6	2,466,362	2,466,558	*SLC9A7*	X	46,623,458	46,624,546	MER21C	LTR/ERVL	X	46,623,722	46,624,477
										*SPRY3*	X	155,794,613	155,795,839	MER21C	LTR/ERVL	X	155,795,271	155,795,776
cfa6_66	*GTF2I*	6	5,671,518	5,671,763						*GTF2IP14*	7	65,095,549	65,096,634	L1MC5a	LINE/L1	7	65,095,820	65,095,976
										*GTF2IP5*	7	65,785,354	65,785,553					

**Table 3:** Dog–human sequence comparison and variations identified by Sanger sequencing in patients and controls. Known and novel (N) SNPs were identified in human *loci*, and common polymorphisms and rare variants were highlighted (MAF = minor allele frequency; MAF > 0.1, common SNP; MAF < 0.1, rare SNP) in the case of those already described. Abbreviation: *n*, number.

Dog *locus*	Gene (Human)	Dog:Human Sequence Comparison Score	Dog:Human Sequence Identity (%)	ASD:CTR Consensus Identity (%)	SNP (N = novel)	Variation	MAF < 0.1 (true = 1; false = 0)	Chr	Start	End	Amplicons Sequenced (ASD)	ASD Carrying Variant		Amplicons Sequenced (CTR)	CTR Carrying Variant	
											*n*	*n*	%	*n*	*n*	%
Cfa6.6	*GALNT17*	100	75.2	98.87	rs1202647	C/T	0	7	71,198,716	71,198,716	10	5	50	10	4	40
					rs10260271	A/G	0	7	71,199,052	71,199,052	10	1	10	10	1	10
Cfa6.7	*NRNX3*	48	78	95												
Cfa6.7	*SPRY3*	32	94.5	99.8	N	G/A		X	155,794,832	155,794,832	10	1	10	10	0	0
					N	G/A		X	155,794,906	155,794,906	10	1	10	10	0	0
					N	G/T		X	155,795,335	155,795,335	10	1	10	10	0	0
					N	C/A		X	155,795,522	155,795,522	10	0	0	10	1	10
Cfa6.7	*SLC9A7*	43	93	97.96	rs1238052830	T/A	1	X	46,623,716	46,623,716	10	0	0	10	1	10
					N	A/G		X	46,623,965	46,623,965	10	0	0	10	1	10
					N	T/C		X	46,624,108	46,624,108	10	0	0	10	1	10
					N	C/T		X	46,624,374	46,624,374	10	1	10	10	0	0
Cfa6.66	*GTF2IP14*	138	76.2	96.58	rs1799101	A/G	0	7	65,095,563	65,095,563	7	7	100	8	8	100
					N	C/A		7	65,095,595	65,095,595	7	0	0	7	1	14
					N	C/T		7	65,095,612	65,095,612	8	4	50	9	5	56
					N	C/T		7	65,095,616	65,095,616	9	2	22	9	2	22
					N	C/T		7	65,095,634	65,095,634	9	1	11	9	0	0
					N	C/T		7	65,095,643	65,095,643	9	0	0	9	1	11
					N	G/A		7	65,095,646	65,095,646	9	0	0	9	1	11
					N	G/A		7	65,095,681	65,095,681	9	8	89	9	9	100
					N	C/A/T		7	65,095,683	65,095,683	9	2	22	9	1	11
					N	T/A		7	65,095,687	65,095,687	9	1	11	9	0	0
					N	T/G		7	65,095,689	65,095,689	9	0	0	9	1	11
					N	T/C		7	65,095,690	65,095,690	9	0	0	9	1	11
					N	T/C		7	65,095,698	65,095,698	9	1	11	9	1	11
					N	AG/CA		7	65,095,709	65,095,710	9	1	11	9	0	0
					N	insGGT		7	65,095,712	65,095,713	9	1	11	9	0	0
					N	T/C		7	65,095,715	65,095,715	9	1	11	9	0	0
					N	C/G		7	65,095,718	65,095,718	9	1	11	9	0	0
					N	T/C		7	65,095,724	65,095,724	9	1	11	9	0	0
					N	insCA		7	65,095,728	65,095,729	9	1	11	9	0	0
					N	A/G		7	65,095,730	65,095,730	9	1	11	9	0	0
					N	T/A		7	65,095,742	65,095,742	9	1	11	9	0	0
					N	C/T		7	65,095,746	65,095,746	9	1	11	9	0	0
					N	C/T		7	65,095,753	65,095,753	9	1	11	9	0	0
					N	insCA		7	65,095,754	65,095,755	9	1	11	9	0	0
					N	A/G		7	65,095,755	65,095,755	9	1	11	9	0	0
					N	C/T/A		7	65,095,765	65,095,765	9	2	22	9	1	11
					N	C/T		7	65,095,778	65,095,778	9	1	11	9	1	11
					N	C/A		7	65,095,779	65,095,779	9	1	11	9	0	0
					N	C/T		7	65,095,833	65,095,833	9	1	11	9	0	0
					N	G/A		7	65,096,185	65,096,185	9	5	56	9	4	44
					rs10262238	C/T	0	7	65,096,197	65,096,197	9	4	44	9	5	56
					N	A/C		7	65,096,281	65,096,281	10	1	10	10	1	10
					N	T/A		7	65,096,298	65,096,298	10	10	100	10	10	100
					rs71562961	A/G	0	7	65,096,467	65,096,467	10	2	20	10	1	10

## Data Availability

Not applicable.
